# Cognitive behavioral therapy for frequent attenders in primary care

**DOI:** 10.1002/hsr2.80

**Published:** 2018-08-18

**Authors:** Ylva Strömbom, Jan Karlsson, Mats Fredrikson, Lennart Melin, Peter Magnusson

**Affiliations:** ^1^ Centre for Research and Development Uppsala University/Region Gävleborg Gävle Sweden; ^2^ Department of Psychology Uppsala University Uppsala Sweden; ^3^ Cardiology Research Unit, Department of Medicine Karolinska Institutet Stockholm Sweden; ^4^ University Health Care Research Center, Faculty of Medicine and Health Örebro University Örebro Sweden; ^5^ Department of Clinical Neuroscience Karolinska Institutet Stockholm Sweden

**Keywords:** applied relaxation, cognitive behavioral therapy, frequent attender, primary care, quality of life, Sweden

## Abstract

**Aim:**

The aim of the study is to investigate if cognitive behavioral therapy given in a group setting affects anxiety and depression, stress, pain, coping strategies during daily life, and health‐related quality of life (HRQoL), among frequent attenders (FAs) in primary care.

**Methods:**

Cognitive behavioral therapy was offered to 331 FAs between 18 and 65 years of age, of whom 89 accepted and 54 completed all steps in the protocol; patients were assigned to 1 of 3 groups: 0, 6, and 12‐month waiting time. The therapy consisted of 12 sessions administered in group format. Outcome measures were Beck's Anxiety Inventory (BAI), Beck's Depression Inventory (BDI), Hospital Anxiety and Depression Scale (HADS), Everyday Life Stress (ELS), Coping Strategy Questionnaire, Multidimensional Pain Inventory‐Swedish version, and Short Form‐36.

**Results:**

Mean age among patients who completed cognitive behavioral therapy was 49.9 years, with a female majority (79.6%). Anxiety and depression scores were reduced after treatment (BAI 16.7 vs 13.6; BDI 16.3 vs 15.7; HADS‐Anxiety 8.41 vs 6.05; HADS‐Depression 7.09 vs 5.69). Because waiting time itself did not affect symptoms, differences reflect treatment effects. Stress ratings were not affected by treatment. Use of nonadaptive coping strategies like *praying and hoping* and *catastrophizing* decreased. Frequent attenders experienced a higher sense of life control. Frequent attenders reported significantly lower HRQoL than general Swedish population norms in all 8 Short Form‐36 domains including mental and physical component summary scores (MCS and PCS), and all domains were unaffected by treatment.

**Conclusion:**

Cognitive behavioral therapy exerts some beneficial effects in FAs. Content of treatment addressed musculoskeletal pain, stress, anxiety, and depression. This broad approach resulted in reduced anxiety, depression, and impact of pain because of enhanced life control.

## 1 INTRODUCTION

Frequent attenders (FA) in primary care consist of 3% to 5% of the general population and use 15% to 25% of available visits to general practitioners (GPs).^1^, ^2^, ^3^ They constitute a heterogenous group, which presents both physical and mental complaints.^4^ Hence, they require substantial resources by presenting diverse symptoms to the GPs.

The psychological factors associated with frequent attendance in primary care are *low* life satisfaction, self‐efficacy, self‐regulation, and self‐esteem as well as *high* perceived stress and negative affect.^1^ In an attempt to address diagnostic diversity and multiple complaits among FAs, it has been suggested that illness behavior should be addressed when considering assessment, course, and treatment.^2^ Illness behavior is described as the varying ways in which individuals define and react to bodily symptoms, experience internal states, make attributions, take actions, and use different kinds of care.^5^ This approach provides a unifying framework when considering otherwise fragmented health‐related information, and is helpful in discriminating therapeutic and prognostic response to a specific treatment among patients with the same diagnosis. Variations in illness behavior interact with life stress, anxiety, and depression,^2^ suggesting that several modulating or mediating variables might be targeted to alter illness behavior and, consequently, frequent attendance.

A review of interventions in primary care in FAs^3^ concluded that a subgroup of depressed patients seem to benefit from a multicomponent depression management programme.^6^, ^7^ These patients reported improved health‐related quality of life (HRQoL) on the SF‐20.^7^ Notably, patients consumed more health care resouces 1 year after intervention, but the increased cost‐effectiveness ratio was about the same as that accepted for other medical treatments.^6^ In an individually formulated cognitive behavioral therapy (CBT), FAs improved their mental component score (MCS) according to Short Form‐36 (SF‐36) at 6 months but reverted at 1 year, while the physical component score (PCS) and measurements of anxiety displayed no change.^8^

It is often not possible to medically account for physical complaints made by FAs. Patients in primary care who suffered from physically unexplained symptoms for more than 2 years were characterized by cognitive deficits, including low attention and slow psychomotor speed, poor verbal skills, and diminished executive function, and they used more nonfunctional passive and avoidant coping strategies than healthy controls.^9^ Coping strategies for pain, anxiety, and stress have been shown to influence HRQoL, perceived impact of pain, and different kinds of psychosocial distress.^10^, ^11^, ^12^, ^13^

Frequent attenders with their various health problems may benefit from CBT using a multimodal approach of applicable methods from relevant areas. However, this approach requires several outcome measurements to evaluate the multiple treatment aspects. In the present study, CBT was based on interventions addressing musculoskeletal pain and psychosocial distress, 2 of the most common reasons for visits to GPs. The aim was to investigate if group CBT would affect the degree of anxiety, depression, stress in everyday life, perceived impact of pain, use of coping strategies, and HRQoL, among FAs in primary care.

## 2 METHODS

### 2.1 Setting

Two health care centers, Björksätra and Vallhov, in Region Gävleborg, Sweden, with a catchment area of 14 000 at the time of data collection, participated. In total, 7 general practitioners and 4 residencies managed patients at the primary care centers.

### 2.2 Frequent attenders

Initially, we selected all (n = 395) FAs between 18 and 65 years with ≥5 face‐to‐face visits to GPs (not other health care providers) during the year 2000. This cutoff value is consistent with previous Swedish studies and mirrors the effect of differences between countries in regard of health care organization and culture.^14^, ^15^, ^16^ A total of 64 FAs were excluded after applying the following criteria: severe mental or somatic disorder, need for an interpreter, ongoing alcohol and/or other drug use disorder, and severe disabilities such as mental retardation. A questionnaire was then sent out to 331 FAs including an offer for CBT treatment. A total of 89 FAs accepted the offer and after being stratified for sex were randomly assigned to 1 of 3 treatment periods (autumn 2001, spring 2002, autumn 2002). A total of 12 treatment sessions were held, and they followed a predefined treatment schedule including questionnaires to be filled out at 4 time points. Questionnaires were sent by regular mail in August (2001 and 2002) and January (2002 and 2003). During CBT, evaluation of current anxiety and depression levels (HADS) was addressed at the beginning of every session.

### 2.3 Dropouts

A total of 54 patients completed all of the steps in the treatment (and all analyses were based on these patients). Of those who discontinued, 14 started treatment, while the remaining 21 announced before treatment started that they would not be able to participate. The patients who discontinued the study provided the following reasons: time shortage, improved health status, family reasons, and group composition. Two patients discontinued without giving any reason.

### 2.4 Data sources and questionnaires

The number of visits to GPs, medication usage, and number of referrals to specialists were collected from medical records. The health problems of the FAs were classified using health complaint categories from medical records based on the patients' own reasons for seeking medical consultation. Health complaints were described and noted by GPs.

Height and weight, number of children at home, university education, employment, use of naturopathic drugs, and alternative medicine were assessed by a questionnaire.

The outcome instruments used were Beck's Anxiety Inventory (BAI), Beck's Depression Inventory (BDI), Everyday Life Stress (ELS), Coping Strategy Questionnaire (CSQ), Multidimensional Pain Inventory‐Swedish version (MPI‐S), SF‐36, and Hospital Anxiety and Depression Scale (HADS), and are described below.

### 2.5 Beck's Anxiety Inventory

This instrument was developed to measure symptoms of anxiety that are largely independent from symptoms of depression. This means that the questionnaire mainly evaluates somatic aspects of anxiety rather than general stress‐related anxiety symptoms,^17^ and a higher score reflects an increased level of anxiety.

### 2.6 Beck's Depression Inventory

This questionnaire was developed^18^ with the purpose of assessing the severity of depression and changes in severity during treatment. It consists of 21 items addressing symptoms and attitudes related to depression such as guilt, pessimism, self‐esteem, social withdrawal, and suicidal thoughts. A higher score suggests a more severe depression.

### 2.7 Everyday Life Stress

ELS consists of 20 statements about stress‐related behavior, including reactions from other people, where a higher score indicates a higher level of stress.^19^ This questionnaire was initially developed for patients with coronary artery disease^20^ but has achieved a widespread use.

### 2.8 Coping Strategy Questionnaire

Coping Strategy Questionnaire assesses coping strategies among patients suffering from long‐lasting pain; 6 cognitive coping strategies (“diverting attention”, “reinterpret pain sensations”, “coping self‐statement”, “ignore pain sensations”, “praying or hoping”, and “catastrophizing”) and 2 behavioral coping strategies (“increased activity level”, “increased pain behaviors”) are adressed. Coping Strategy Questionnaire measures the frequency and success of coping strategies, and has been validated in Swedish spinal pain patients.^21^, ^22^ A higher score indicates frequent use of a specific coping strategy.

### 2.9 Multidimensional Pain Inventory‐Swedish version

Multidimensional Pain Inventory‐Swedish version is primarily used to identify patients with well‐functioning or dysfunctional coping strategies developed for Swedish usage^23^, ^24^ based on a modified version of West Haven‐Yale Multidimensional Pain Inventory.^25^ We used parts 1 and 2 (but excluded part 3) of MPI‐S, which measure psychosocial and behavioral consequenses of coping strategies among individuals with long‐lasting pain. Part 1 subscales are life interference, support, life control, pain severity, and affective distress, while the second part covers distracting, negative, and solicitous responses from significant others. A lower score implies lower self‐reported influence of the impact of a specific subscale; a higher score implies a higher impact.

### 2.10 Short Form health survey (SF‐36) domains and general Swedish population norms

The generic HRQoL instrument SF‐36 consists of 36 items and measures 8 domains: physical functioning (PF), role physical (RP), bodily pain (BP), general health (GH), vitality (VT), social functioning (SF), role emotional (RE), and mental health (MH). A higher score on the 0 to 100 scale indicates better HRQoL. In addition, the PCS score and the MCS score, calculated from the 8 domains, are norm‐based with a mean of 50 in the general population.^26^

### 2.11 Hospital Anxiety and Depression Scale

This questionnaires' purpose is to gain knowledge of anxiety and depression among patients treated within somatic health care in the absence of psychiatric specialists.^27^ It is a screening instrument, not used for diagnostic purposes. Hospital Anxiety and Depression Scale consists of 14 questions addressing anxiety symptoms and symptoms of depression (7 questions each). Anxiety and depression are scored separately, and a high score indicates the presence of anxiety and/or depression.^28^

### 2.12 Cognitive behavior therapy

The treatment was performed in a group setting with a maximum of 8 patients per group. The content was based on a review of the medical records reflecting patients' health complaint categories including musculoskeletal pain, infections, psychosocial distress, digestive problems, skin complaints, injuries, headache and tinnitus complaints, genitor urinary complaints, circulatory complaints, and respiratory complaints.

Cognitive behavior therapy was given during 12 sessions in total, including maintenance of learned skills and a booster (follow‐up) session. Each 2‐hour session began with a mindfulness exercise followed by a lecture on the topic of the session and by a group discussion. The topics were administered in the same order, and included the same content for all groups. Topics included were physical and psychological consequences of pain and stress, health awareness (including information about tobacco, alcohol, and diet), and coping strategies (problem solving, scheduling, distraction techniques, and cognitive restructuring), followed by instruction for maintaining skills and a booster session. Each session ended with applied relaxation, a coping technique that teaches patients to relax rapidly when signs of anxiety occur in everyday life. Applied relaxation has been proven effective for control of various anxiety and somatic and psychosomatic disorders.^29^, ^30^, ^31^ At the end of each session, homework assignments were given, mainly focused not only on applied relaxation techniques but also on tasks related to the session topic. In addition, participants were asked to keep a diary containing information regarding exercise, food intake, adjustments in medication, and stressful life events.

### 2.13 Ethics

The Gävle‐Dala research ethics committee approved the study (Dnr 2000346), and each patient gave a written informed consent.

### 2.14 Statistics

Descriptive data are presented as numbers, percentages, and means, including standard deviations and 95% confidence intervals. The first questionnaire set was used as a baseline.

The random intercept model was used to evaluate effects of treatment on questionnaire scores (except for SF‐36 domains). The study design with 3 treatment periods allows for comparisons between treated and not‐yet‐treated subjects within 1 model, and provides treatment effects adjusted for the time between study entry and treatment. Model‐based standarized effect sizes for within‐subject effects were calculated by dividing the absolute treatment effect by the within‐subject (residual) standard deviation.^32^ We chose the random intercept mixed model because it allows testing for both time and treatment effects, in situations with repeated measurements.

Comparisons of SF‐36 domains were done with the nonparametric Mann‐Whitney *U* test for between groups, and with the Wilcoxon signed‐rank test for paired data. Standarized effect sizes for comparisons of SF‐36 domains between groups were estimated using Cohen *d* and using standardized response mean (SRM), for change between baseline and postintervention. Standardized effect sizes were interpreted as follows: trivial (<0.20), small (0.20‐0.49), moderate (0.50‐0.79), and large (≥0.80).

In all statistical analyses, a 2‐sided *P* value lower than 0.05 was considered statistically significant. Because of the semiexploratory nature of this study, we choose not to adjust for multiple testing. This should be keept in mind when interpreting the results. All statistical analyses were performed using SPSS version 22 (IBM, Armonk, NY).

## 3 RESULTS

### 3.1 Patient characteristics

Patient characteristics, including health complaints, are summarized in Table 1. Mean age was 49.7 (SD 11.1) years with a female majority n = 43 (79.6%). The 3 most common health complaint categories were musculoskeletal pain, infections, and psychosocial distress.

**Table 1:** Patient characteristics at baseline

Variable	Total n = 54 (%)
Female sex	43 (79.6%)
Married/cohabitate	45 (83.3%)
Child(ren) at home	18 (33.3%)
University	4 (7.4%)
Employed	35 (64.8%)
Body mass index ≥30 kg/m^2^	12 (22.2%)
Naturopathic drug use	12 (22.2%)
Alternative medicine use	18 (33.3%)
Health complaint categories	
Musculoskeletal pain	42 (77.8%)
Infections	39 (72.2%)
Psychosocial distress	29 (53.7%)
Digestive problems	26 (48.1%)
Skin complaints	19 (35.2%)
Injuries	12 (22.2%)
Headache and tinnitus complaints	16 (29.6%)
Genitor urinary complaints	13 (24.1%)
Circulatory complaints	13 (24.1%)
Respiratory complaints	16 (29.6%)

### 3.2 Health care‐related outcome measurement

No difference regarding number of visits to GPs was noted between baseline and after treatment.

At baseline, 10 patients used antidepressant medication and 3 additional patients received antidepressive treatment during the study period. The use of benzodiazepines and morphine‐based pain medication showed similar patterns. Only 1 patient used benzodiazepine medication continually, and 7 had temporary prescriptions. Morphine‐based pain medications were continually used by 3 patients, while 8 had temporary prescriptions.

The number of referrals from primary care to specialist care showed no difference during the study period (baseline vs after treatment).

### 3.3 BAI and BDI

Beck's Anxiety Inventory and BDI results are summarized in Tables 2 and 3. The mean score of BAI at baseline was 16.7 (SD 9.5) and after treatment, 13.6 (SD 7.0); thus, a moderate reduction (ES = 0.70) of anxiety symptoms (*P* = .001) occurred. There was a significant (*P* = .022) but small reduction (ES = 0.46) in depression symptoms according to BDI (16.3, SD 9.5 vs 15.7, SD 9.8). The variable time did not affect outcome. The improvement was consistent over time for 6 and 12 months respectively (*P* = .001). Patients did not improve during the waiting period (6 and 12 months respectively), supporting that treatment results could not be attributed to the mere passage of time or repeated measurements.

**Table 2:** Questionnaire (BAI, BDI, ELS, CSQ, MPI‐S) scores in frequent attenders in primary care at baseline and posttreatment

	Baseline (n = 54)	Posttreatment
Questionnaire	Mean (SD)	Mean (SD)
Becks Anxiety Inventory (BAI)	16.7 (9.5)	13.6 (7.0)
Becks Depression Inventory (BDI)	16.3 (9.5)	15.7 (9.8)
Everyday Life Stress (ELS)	22.6 (11.0)	21.6 (10.5)
Coping Strategy Questionnaire (CSQ)		
Diverting attention	10.3 (8.0)	11.0 (8.4)
Reinterprating pain sensations	5.0 (5.4)	4.8 (4.8)
Coping self‐statements	14.4 (7.9)	14.6 (7.3)
Ignoring sensations	11.1 (7.0)	11.2 (6.7)
Praying/hoping	9.9 (7.5)	8.2 (7.3)
Catastrophizing	12.5 (9.3)	10.2 (7.9)
Increased behavioral activities	13.5 (7.7)	12.7 (7.2)
Pain behaviors	13.5 (7.2)	14.6 (7.3)
Multidimensional Pain Inventory (MPI‐S)		
Pain impact		
Life interference	2.7 (1.7)	2.4 (1.7)
Support	2.8 (1.8)	2.8 (1.8)
Life control	2.5 (1.7)	2.9 (1.7)
Pain severity	2.8 (1.7)	2.6 (1.7)
Affective distress	2.8 (1.2)	2.4 (1.1)
Responses by significant others		
Distracting responses	1.7 (1.5)	1.6 (1.4)
Negative responses	1.0 (1.3)	0.9 (1.3)
Solicitous responses	2.3 (1.7)	2.0 (1.5)

Abbreviation: SD, standard deviation.

**Table 3:** Estimated effect sizes of cognitive behavioral therapy for frequent attenders in primary care

Questionnaire	Estimated Change	Within‐Subject SD	95% CI	Standardized Effect Size^b^	*P* Value^a^
Becks Anxiety Inventory (BAI)	−3.67	4.66	−5.72 to −1.62	0.702	.001
Becks Depression Inventory (BDI)	−2.12	4.60	−3.93 to −0.32	0.462	.022
Everyday Life Stress (ELS)	−1.52	4.48	−3.28 to 0.24	0.339	.090
Coping Strategy Questionnaire (CSQ)					
Diverting attention	0.96	4.31	−0.70 to 2.63	0.224	.250
Reinterprating pain sensations	0.26	2.84	−0.84 to 1.36	0.091	.637
Coping self‐statements	0.59	4.78	−1.25 to 2.44	0.124	.522
Ignoring sensations	0.17	4.51	−1.58 to 1.91	0.037	.849
Praying/hoping	−1.98	4.17	−3.59 to −0.37	0.475	.017
Catastrophizing	−2.46	5.15	−4.45 to −0.48	0.479	.016
Increased behavioral activities	0.33	4.35	−1.35 to 2.01	0.077	.692
Pain behaviors	1.78	3.62	0.39 to 3.18	0.491	.014
Multidimensional Pain Inventory (MPI‐S)					
Pain impact					
Life interference	−0.21	0.87	−0.55 to 0.13	0.242	.218
Support	0.22	1.07	−0.19 to 0.63	0.208	.284
Life control	0.64	1.09	0.22 to 1.06	0.587	.004
Pain severity	−0.10	1.02	−0.50 to 0.29	0.103	.595
Affective distress	−0.34	0.92	−0.70 to 0.02	0.369	.060
Responses by significant others					
Distracting responses	0.02	1.02	−0.38 to 0.41	0.018	.925
Negative responses	−0.06	0.99	−0.44 to 0.33	0.072	.771
Solicitous responses	−0.08	1.15	−0.53 to 0.36	0.056	.709

Abbreviations: CI, confidence interval; SD, standard deviation.

a Estimates and *P* values obtained by random intercept models.

b Standardized by within‐subject SD: estimated change/SD.

### 3.4 ELS, CSQ, and MPI‐S

As shown in Table 3, ELS showed a tendency toward reduced stress symptoms, with a small ES. This improvement was most pronounced after 6 months; conversely, waiting for treatment had no effect on outcome.

Coping Strategy Questionnaire scores after treatment were significantly changed in 3 of the 8 subscales (Table 3) indicating reduced use of the coping strategies *praying/hoping* and *catastrophizing* but an increase in *pain behavior*. Notably, *pain behavior* was more frequently used during the waiting period and did not change after treatment. *Catastrophizing* was less often used after treatment, indicating an improvement.

The MPI‐S score showed no significant difference after treatment with 1 exception; life control had a significant improvement with a moderate ES. Multidimensional Pain Inventory‐Swedish version is summarized in Table 3.

### 3.5 SF‐36

Treatment did not affect outcome with regard to SF‐36. Neither waiting for treatment nor time after treatment had an effect on HRQoL. Frequent attenders in this cohort were compared to Swedish population norms. In all 8 domains and both MCS and PCS, mean scores were significantly worse and the ES was large. After treatment, there was no difference compared to baseline. Short Form‐36 is summarized in Table 4.

**Table 4:** SF‐36 scores in frequent attenders in primary care at baseline compared to general Swedish population norms and postintervention

	Frequent Attenders		Swedish Population Norms (n = 1524)		Frequent Attenders vs Norms^a^	Frequent Attenders		Frequent Attenders Baseline vs Postintervention^b^
	Baseline					Postintervention		
	n = 54					n = 54		
SF‐36 Domains	Mean (SD)	95% CI	Mean (SD)	95% CI	Effect Size	Mean (SD)	95% CI	Effect Size
Physical functioning	67.2 (23.8)	60.5‐73.9	87.1 (18.5)	86.2‐88.1	0.93	67.2 (25.7)	60.0‐74.5	0.00
Role physical	52.5 (43.4)	40.3‐64.7	82.0 (32.4)	80.4‐83.7	0.77	47.6 (43.7)	35.3‐59.8	−0.17
Bodily pain	49.1 (25.8)	41.9‐56.4	72.1 (26.8)	70.8‐73.5	0.87	48.4 (27.2)	40.8‐56.1	−0.09
General health	48.8 (24.5)	41.9‐55.8	73.2 (23.2)	72.0‐74.4	1.02	48.0 (22.2)	41.8‐54.3	−0.08
Vitality	41.0 (26.5)	33.7‐48.4	67.7 (23.1)	66.6‐68.9	1.07	47.3 (24.1)	40.5‐54.0	0.28
Social functioning	64.2 (27.2)	56.6‐71.8	88.2 (20.2)	87.2‐89.2	1.00	67.7 (25.5)	60.5‐74.8	0.14
Role emotional	52.7 (45.2)	39.8‐65.5	86.1 (28.1)	84.7‐87.6	0.89	58.2 (43.6)	45.9‐70.4	0.11
Mental health	59.1 (21.9)	53.0‐65.2	80.0 (19.1)	79.0‐80.9	1.02	63.0 (19.1)	57.7‐68.4	0.20
Physical component summary	40.6 (11.6)	37.3‐44.0	49.5 (9.6)	49.0‐50.0	0.84	38.3 (12.1)	34.9‐41.7	−0.21
Mental component summary	38.2 (13.6)	34.2‐42.1	50.0 (10.2)	49.4‐50.5	0.98	41.1 (12.2)	37.7‐44.5	0.21

Effect size: standardized response mean.

Abbreviations: CI, confidence intervall; SD, standard deviation.

a Frequent attenders vs norms: all comparisons significant (Mann‐Whitney *U* test, *P* < .0001); effect size: Cohen *d*.

b Frequent attenders baseline vs postintervention: all comparisons nonsignificant (Wilcoxon signed‐rank test).

### 3.6 HADS

**Figure 1:**
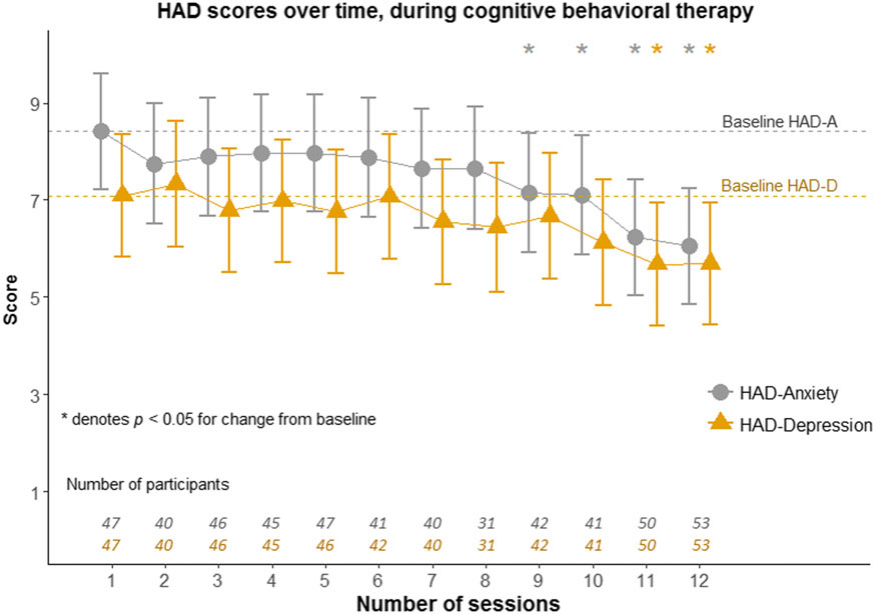
Development over time of Hospital Anxiety and Depression Scores (HADS) measured session by session. Scores are presented as mean values including 95% confidence interval. The *P* value is calculated from the random intercept model

Cognitive behavioral therapy significantly lowered anxiety and depression scores as measured with HADS from sessions 9 and 10 respectively. This effect remains at follow‐up after 3 months after intervention (Figure 1).

## 4 DISCUSSION

The health complaints targeted for CBT in the present study were musculoskeletal pain and psychosocial distress, which was defined as subjective reports of stress, anxiety, and depression; these were the most common reasons among FAs for visits to GPs. In the present study, CBT reduced anxiety and depression, increased life control, and reduced the use of the coping strategies of “praying and hoping” and “catastrophizing”. Health‐related quality of life, measured with the generic questionnaire SF‐36, was not significantly affected by treatment.

### 4.1 Interpretation of results

The levels of anxiety and depression reported by FAs in the current study was mild to moderate at baseline, but was further reduced as a function of treatment. The effects might seem moderate, but these findings are reliable because they cross‐validate over 2 independent measures of anxiety and depression: BAI and BAI on the one hand and HADS on the other.

Taking into account the mild to moderate levels of anxiety and depression displayed, it can be argued that FAs are correct in that psychosocial factors are not the main reason they need help from GPs.^4^ Anxiety and depression might simply be the consequences of other health issues, and if the cause of despair is managed, symptoms may vanish without any CBT interventions. Also, if anxiety and depression are considered a result of maladaptive illness behavior, that would explain why all symptoms do not apply to all patients with the same diagnosis, and why symptom alleviation is reached by different interventions for different individuals. The anxiety reduction after intervention found in our sample may partly be explained by the use of alternative coping methods as well as by the applied relaxation intervention.

Impact of pain was indeed reduced, which in part may explain the reduced anxiety and depression after CBT. Less life interference and affective distress, along with a higher degree of life control, could improve patients' life situations.

In our study, FAs presented several concurrent medically unexplained complaints, and most had previously sought help on several occasions without sufficient symptomatic relief. The use of CBT with a focus on the 2 most prevalent complaint categories resulted in lowered anxiety and depression, the perception of a somewhat reduced impact of pain on life, and a few altered coping strategies, but no change in generic HRQoL. Short Form‐36 is a widely used generic instrument that is not disease specific. In the present study, there was no change in HRQoL after CBT. However, SF‐36 is probably not sensitive enough to measure these changes, and important aspects of FAs' complaints may not be completely covered. Instead, additional outcome measurements may be needed to evaluate and judge the efficacy of CBT for FAs.

### 4.2 Other studies

Cognitive behavioral therapies have been proven effective for a number of diagnoses, including anxiety and pain.^33^ In this study, lower anxiety and depression ratings were shown as results of CBT. We demonstrated that CBT in a group setting, including psychological as well as pain‐control interventions in the same treatment program, was useful for FAs. Interventions are most often chosen to address specific symptoms or conditions, but here, we tried an overall approach. Frequent attenders differ from other patient groups in their demand for health care services, including staff time, and they are hard to sort into diagnostic categories.^34^The extent of CBT was, in part, determined by the applied relaxation training manual and seems to be beneficial for this particular patient group. Applied relaxation, the base ingredient in the present study, originates from behavioral therapy, and its practice leads to reduced pain and anxiety, an increase in perceived control, and reduced symptoms.^29^, ^30^, ^31^, ^35^ This is consistent with our results. In previous studies, psychological factors have been of great interest when trying to explain the excessive help‐seeking pattern among FAs.^36^ However, in at least 1 study, FAs did not agree with the statement that psychological factors explained their malaise.^4^

Coping strategies for pain changed as a function of CBT, and these changes reflect less use of passive coping strategies such as *catastrophizing* and *praying and hoping*. In previous research, catastrophizing was associated with low ratings of self‐efficacy, whereas hoping and praying were associated with a perceived external focus, ie, reasons for suffering are attributed to factors outside of the individual. Catastrophizing was also related to poorer emotional adjustment with increased anxiety and depression.^21^, ^37^ Our results indicate an increased sense of internal control and self‐efficacy, but this need to be examined in more detail.

Musculoskeletal pain and psychosocial distress are both known to affect HRQoL. Frequent attenders included in the study rated their HRQoL worse than the Swedish norm population on all SF‐36 domains. This is conceptually consistent with previous findings, even though different outcome measurements have been used.^35^, ^38^, ^39^, ^40^ Although we found no improvement of HRQoL in the present study, 1 previous multicomponent intervention including assessment, medication, education, monitoring, and evaluation showed HRQoL improvement in FAs suffering from depression.^7^ The improved outcome from using such an approach is likely because of several factors, including continuous physician contact and the fact that the intervention involved the patients' own GPs.

Most FAs are content with their GP,^38^ and the impact of trust and contentment with health care providers is crucial for successful outcomes when it comes to patients who suffer from the kinds of illness that necessitate frequent medical consultations. The combination of psychological and medical staff involved in CBT in the present study contributes to increased impact of this treatment rationale. Indeed, this combination approach was identified in a study on individual CBT as being an important influence on the visiting patterns of FAs.^8^

### 4.3 Limitations of the study

The selection of patients deserves special attention, because they were invited in a research context to take part in a CBT group therapy administered by a single health care provider. Also, long‐term results beyond the follow‐up period remain unknown. Furthermore, the CBT concept is broad, and specific applications vary, which may hamper generalization of these results. In addition, the heterogeneous group of FAs may possibly benefit more from individualized CBT and disease‐specific management programs including multiprofessional teams addressing both physical and psychological components of the illness at hand. It is unknown if health complains and/or organization of health care in this study have changed over time. This study is limited by the small sample size; statistical hypothesis testing may be subject to type II errors. Future studies on FAs may include larger patient groups (preferably with predefined degree of symptoms) to allow for randomization to different treatment modalities given in‐group or individually. Another interesting approach would be to include FAs with varying health issues and randomize them into treatment primarily addressing either physical illness or psychological distress.

## 5 CONCLUSIONS

Cognitive behavioral therapy given in a group setting results in decreased feelings of anxiety and depression in the short term among FAs, and effects remain durable over a time period of 1 year. Group CBT changed the impact of pain on their life, reflecting improved life control. Nonadaptive coping strategies, ie, praying and hoping and catastrophizing, decreased. The results imply that broad treatment content is useful for a heterogeneous group of FAs. Thus, FAs benefit from CBT given in a group setting.

## FUNDING

The Region Gävleborg and FINSAM funded this research project.

## CONFLICT OF INTEREST

No conflicts of interest declared.

## AUTHOR CONTRIBUTIONS

Conceptualization: Ylva Strömbom, Lennart Melin

Formal analysis: Ylva Strömbom, Jan Karlsson, Mats Fredrikson, Lennart Melin, Peter Magnusson

Funding acquisition: Ylva Strömbom

Writing—review and editing: Ylva Strömbom, Jan Karlsson, Mats Fredrikson, Lennart Melin, Peter Magnusson

Writing—original draft: Ylva Strömbom, Peter Magnusson

## Supporting information

Data S1 Supporting information item

Data S2
Supporting information item
