# Supplementary material for: Cognitive behavioral therapy for frequent attenders in primary care
Source: Health Sci Rep. 2018 Aug 18;1(9):e80. doi: 10.1002/hsr2.80 (PMC6266570; doi:10.1002/hsr2.80)
Supplement: Supplementary file 1 — Data S1 Supporting information item [file HSR2-1-e80-s001.docx]

| **Participant Flow Chart** |
| --- |
|  |
| Identified Frequent attenders* (n=395) |
| 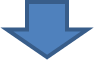   \|  \| \| --- \| |
| Exclusion (n=64) Reasons: severe mental or somatic disorder, need for interpreter, on-going alcohol or drug abuse, severe disabilities such as mental retardation. |
| 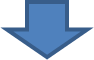   \|  \| \| --- \| |
| Invitation to participate (n=331) |
| 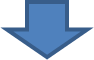   \|  \| \| --- \| |
| Consent to participate (n=89) |
| 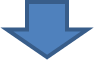   \|  \| \| --- \| |
| Cancellation before treatment started (n=21) |
| Group 1: n=7 , group 2: n= 5, group 3: n=11 |
|  |
| Discontinued participation (n=14) |
| Group 1: n=4, group 2: n=4, group 3: n=2 |
|  |
| Reasons: time shortage, improved health, family reasons, and group composition. |
| 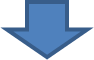   \|  \| \| --- \| |
| Completion of the study (n=54) |
| Group 1: n=17, group 2: n=20, group 3: n=17 |
|  |
| * A coordinate system using number of patients (x-axis) and number of visits (y-axis) was used to define frequent attenders. Vectors meeting point was the border between frequent and regular visiting. Frequent attenders were defined as patients who visited GPs ≥5 time during the study year (7.5% of the patients at the health care centers). |

| **Study design** | | |  |  |  |  |  |  |  |  |  |  |  |  |  |  |  |  |  |
| --- | --- | --- | --- | --- | --- | --- | --- | --- | --- | --- | --- | --- | --- | --- | --- | --- | --- | --- | --- |
|  |  |  |  |  |  |  |  |  |  |  |  |  |  |  |  |  |  |  |  |
| Group 1, n=17 |  | Cognitive Behavior Therapy | | | | |  |  |  |  |  |  |  |  |  |  |  |  |  |
| Group 2, n=20 |  |  |  |  |  |  |  | Cognitive Behavior Therapy | | | | |  |  |  |  |  |  |  |
| Group 3, n=17 |  |  |  |  |  |  |  |  |  |  |  |  |  | Cognitive Behavior Therapy | | | | |  |
| Questionnaire | Q1 |  |  |  |  |  | Q2 |  |  |  |  |  | Q3 |  |  |  |  |  | Q4 |
| Months | 0 | 1 | 2 | 3 | 4 | 5 | 6 | 7 | 8 | 9 | 10 | 11 | 12 | 13 | 14 | 15 | 16 | 17 | 18 |
|  |  |  |  |  |  |  |  |  |  |  |  |  |  |  |  |  |  |  |  |
| A total of 54 frequent attenders, randomly assigned into 3 groups, underwent Cognitive Behavioral Therapy (CBT) and were evaluated using a package (Beck’s Anxiety Inventory (BAI), Beck’s Depression Inventory (BDI), Coping Strategy Questionnaire (CSQ), Everyday Life Stress (ELS), *Multidimensional Pain Inventory- Swedish version (*MPI-S), Short Form health survey (SF-36)) of questionnaires (Q) at 4 times to all patients (Q1, Q2, Q3, Q4) over a period of 18 months. Group 1 received CBT following Q1 while Group 2 received after 6 months and Group after 12 months, respectively. HADS was administrated at the start of each session. CBT was performed as 12 sessions over a 5 month period. | | | | | | | | | | | | | | | | | | | |
