# Supplementary material for: Cognitive behavioral therapy for frequent attenders in primary care
Source: Health Sci Rep. 2018 Aug 18;1(9):e80. doi: 10.1002/hsr2.80 (PMC6266570; doi:10.1002/hsr2.80)
Supplement: Supplementary file 2 — Data S2 Supporting information item [file HSR2-1-e80-s002.docx]

**Description of Cognitive Behavioral Therapy applied to frequent attenders.**

Treatment was performed in a group setting with a maximum of eight patients per group. Cognitive behavioral therapy (CBT) was given during a total of 12 sessions, including maintenance of learned skills and a booster (follow-up) session. Each two-hour session began with square breathing, a mindfulness exercise aimed to increase focus on the topic of the day. To keep track of participants and increase probability of detecting mood swings during the whole treatment period HADS was used as screening instrument.

CBT content was based on the two most common health complaints among frequent attenders, musculoskeletal pain and psychosocial distress, and operationalized as pain, stress, anxiety and depression.

Treatment was administered in the same order, and included the same content for all groups. Each session ended with applied relaxation training. At the end of treatment maintenance of required skills was discussed and individualized. The final session was in part given to each participant individually and included their HADS results over time and a discussion about what ingredient was helpful to that person. Individual maintenance strategies was addressed and adjusted.

**Session 1.**

- Information regarding the present study.
- Review and completion of questionnaires.
- Introduction to applied relaxation training including related homework assignment.
 **Session 2.**

- Topic of the session was bodily reactions to pain including anatomy and physiology.

- Discussion regarding the topic of the session related to individual experiences in the group.
- Applied relaxation training including related homework assignment.

**Session 3.**
- Topic of the session was impact of pain on everyday life, including anxiety and depression related to pain. Behavior experiments designed to challenge pain behavior were introduced.

- Discussion regarding the topic of the session related to individual experiences in the group.
- Applied relaxation training including related homework assignment. In addition, participants were given tasks related to strategies discussed. The latter was optional.

**Session 4**.
- Topic of the session was reactions to stress related to the hormone system of the body, including related anatomy and physiology.
- Discussion regarding the topic of the session related to individual experiences in the group.
- Applied relaxation training including related homework assignment.

**Session 5.**
- Topic of the session was consequences of stress in everyday life, including anxiety and depression symptoms. Cognitive restructuring were introduced.

- Discussion regarding the topic of the session related to individual experiences in the group.
- Applied relaxation training including related homework assignment. In addition, participants were given tasks related to strategies discussed. The latter was optional.

**Session 6**.
- Health behavior including health awareness. Brief information about effects of smoking, alcohol, drugs, food intake (i.e. overeating) and exercise was given. Strategies for behavior changes in related areas present among participants in group were described.

- Discussion regarding the topic of the session related to individual experiences in the group.
- Applied relaxation training including related homework assignment. In addition, participants were given tasks related to behavior change strategies discussed, which was optional.

**Session 7**.
- Health care priority system and how patients can help GPs making optimal assessments.

- Information and strategies needed to enhance GPs assessments was addressed.

- Discussion regarding the topic of the session related to individual experiences in the group.
- Applied relaxation training including related homework assignment.

**Session 8**.
- Coping strategies addressing pain, including descriptions of security and avoidance behaviors. This session included problem solving, time planning, distraction techniques related to pain as well as cost benefit reasoning.

- Discussion regarding the topic of the session related to individual experiences in the group.
- Applied relaxation training including related homework assignment. In addition, participants were given tasks related to strategies discussed. The latter was optional.

**Session 9**.
- Coping strategies addressing stress, anxiety and depression, including security and avoidance behaviors. This session work regarding problem solving and time planning continued. Behavioral strategies such as activation, exposure to perceived anxiety triggers and strategies to handle stressful situations were introduced.

- Discussion regarding the topic of the session related to individual experiences in the group.
- Applied relaxation training including related homework assignment. In addition, participants were given tasks related to strategies discussed. The latter was optional.

**Session 10**.
- Mindfulness as coping strategy to prevent rumination about past events or worry about the future. These tools are mainly focused on what is going on in the present.

- Discussion regarding the topic of the session related to individual experiences in the group.
- Applied relaxation training including related homework assignment. In addition, participants were given tasks related to strategies discussed. The latter was optional.

**Session 11**.
- Follow-up including development of individual maintenance strategies derived from previous content practice and discussions.

**Session 12**.
- Follow-up session including individual feedback regarding HADS results and an opportunity for patients to ask questions about treatment in regard to their personal situation.
